# 

**Published:** 1858-05

**Authors:** 


### CONTENTS.

### *ORIGINAL COMMUNICATIONS.*

PAGE.

ARTICLE I.—Chlorate of Potassa. By T. S. WEATHERLY, M. D., Montgomery, Alabama,........................................................ 515

ARTICLE II.—The Heart. By HORATIO N. HOLLIFIELD, A. M., M. D., Sandersville, Georgia,........................................................ 516

ARTICLE III.—Cases showing the advantage of heroic doses of Calomel. By WM. HAUSER, M. D., of Jefferson county, Georgia,........................................ 518

### *SELECTIONS.*

Medical Societies and Clinical Reports—Philadelphia County Medical Society, 522

Valedictory Address. By OLIVER WENDELL HOLMES, M. D.,...................... 530

Report to the State Society by Committee on Rheumatism,........................ 541

Extracts from the Transactions of the Baltimore Pathological Society,........... 549

On the Injection of Urea and other Substances into the Blood,.................. 560

### *EDITORIAL AND MISCELLANEOUS.*

Course of Lectures for 1856........................................................ 568

Bibliographical,........................................................ 568

Annual Address before the Medical Society of Georgia,........................ 570

Medical and Surgical Reporter,........................................................ 570

Oglethorpe Medical and Surgical Journal,........................................ 571

Medical Society of the State of Georgia,........................................ 571

Journal of Materia Medica and Pharmaceutic Formulary,........................ 577

Paris Hospitals,........................................................578

